# (Aceto­nitrile-κ*N*)iodidobis(tri­phenylphosphane-κ*P*)copper(I)

**DOI:** 10.1107/S1600536814010940

**Published:** 2014-05-17

**Authors:** Yupa Wattanakanjana, Arunpatcha Nimthong, Jedsada Mokakul, Phattarin Sukpornsawan

**Affiliations:** a Department of Chemistry, Faculty of Science, Prince of Songkla University, Hat Yai, Songkhla 90112, Thailand

## Abstract

In the mononuclear title complex, [CuI(CH_3_CN)(C_18_H_15_P)_2_], the Cu^I^ ion is in a distorted tetra­hedral geometry, coordinated by two P atoms of two tri­phenyl­phosphane ligands, one N atom of an aceto­nitrile ligand and one iodide anion. The aceto­nitrile ligand is disordered over two sets of sites in a 0.629 (15): 0.371 (15) ratio. In the crystal, weak C—H⋯I hydrogen bonds link the mol­ecules, forming a chain along [100].

## Related literature   

For potential applications of copper(I) complexes, see: Tian *et al.* (2004 ▶); Krupanidhi *et al.* (2008 ▶); Aslanidis *et al.* (2010 ▶); Gallego *et al.* (2012 ▶). For related structures, see: Balili & Pintauer (2007 ▶); Royappa *et al.* (2013 ▶).
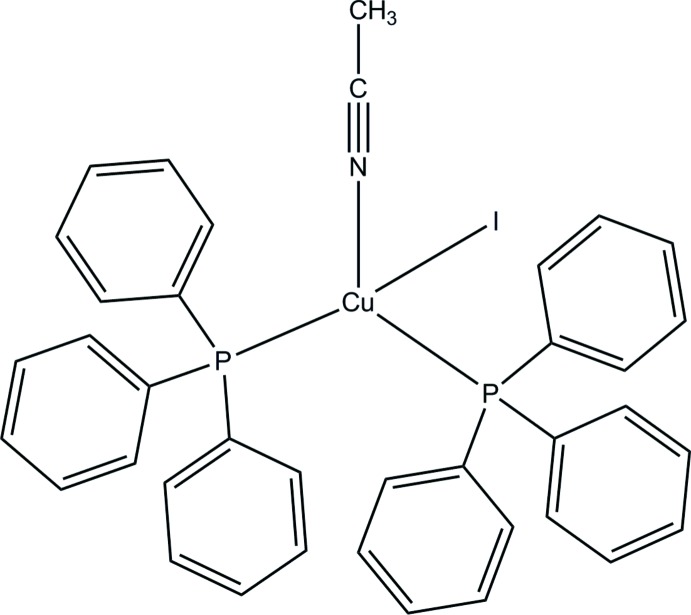


## Experimental   

### 

#### Crystal data  

[CuI(C_2_H_3_N)(C_18_H_15_P)_2_]
*M* *_r_* = 756.03
Monoclinic, 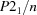

*a* = 9.2547 (3) Å
*b* = 19.3814 (6) Å
*c* = 19.4249 (6) Å
β = 93.043 (1)°
*V* = 3479.31 (19) Å^3^
*Z* = 4
Mo *K*α radiation
μ = 1.63 mm^−1^
*T* = 100 K
0.33 × 0.22 × 0.09 mm

#### Data collection  

Bruker AXS SMART APEX CCD diffractometer
Absorption correction: multi-scan (*SADABS*; Bruker, 2012 ▶) *T*_min_ = 0.600, *T*_max_ = 0.746
20389 measured reflections
10066 independent reflections
8394 reflections with *I* > 2σ(*I*)
*R*_int_ = 0.025

#### Refinement  

*R*[*F* ^2^ > 2σ(*F* ^2^)] = 0.036
*wR*(*F* ^2^) = 0.086
*S* = 1.09
10066 reflections
400 parameters
3 restraints
H-atom parameters constrained
Δρ_max_ = 1.59 e Å^−3^
Δρ_min_ = −0.52 e Å^−3^

### 

Data collection: *APEX2* (Bruker, 2012 ▶); cell refinement: *SAINT* (Bruker, 2012 ▶); data reduction: *SAINT*; program(s) used to solve structure: *SHELXS97* (Sheldrick, 2008 ▶); program(s) used to refine structure: *SHELXL2012* (Sheldrick, 2008 ▶) and *SHELXLE* (Hübschle *et al.*, 2011 ▶); molecular graphics: *Mercury* (Macrae *et al.*, 2008 ▶); software used to prepare material for publication: *SHELXL97* (Sheldrick, 2008 ▶) and *publCIF* (Westrip, 2010 ▶).

## Supplementary Material

Crystal structure: contains datablock(s) I. DOI: 10.1107/S1600536814010940/lh5703sup1.cif

Structure factors: contains datablock(s) I. DOI: 10.1107/S1600536814010940/lh5703Isup2.hkl

CCDC reference: 1002549

Additional supporting information:  crystallographic information; 3D view; checkCIF report

## Figures and Tables

**Table 1:** Hydrogen-bond geometry (Å, °)

*D*—H⋯*A*	*D*—H	H⋯*A*	*D*⋯*A*	*D*—H⋯*A*
C2—H2*A*⋯I1^i^	0.98	3.09	3.727 (8)	124

Symmetry code: (i) 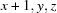.

**Fig. 1.**
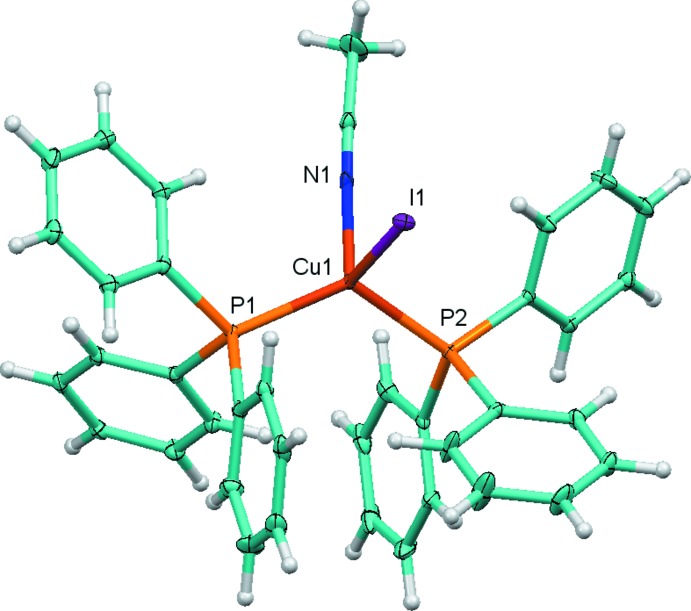
The molecular structure of the title compound with displacement ellipsoids shown at the 30% probability level. The disorder is not shown.

**Fig. 2.**
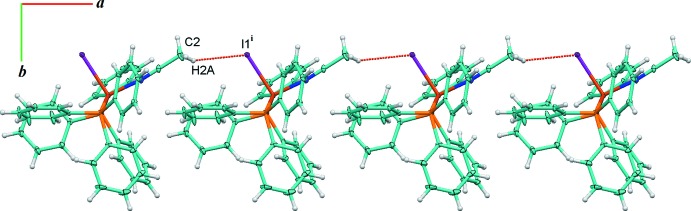
Part of the crystal structure showing weak intermolecular C—H···I hydrogen bonds (dashed lines), leading to the formation of a 1-D chain along the a-axis direction (symmetry code (i): x+1, y, z).

## supplementary crystallographic information

### 1. Experimental

Tri­phenyl­phosphane (0.14g, 0.5 mmol) was dissolved in 30 cm^3^ of aceto­nitrile in a round flask equipped with reflux condenser and magnetic stirrer at 335 K. CuI (0.10g, 0.5 mmol) was added and the mixture was stirred for 6 hrs. Solid 5-amino-1,3,4-thia­diazole-2-thiol (0.07 g, 0.05 mmol) was added and the new reaction mixture was heated under reflux for 8 hrs where upon the precipitate gradually disappeared. The resulting clear solution was filtered and left to evaporate at room temperature. Colorless crystals, which deposited upon standing for several days, were filtered off, washed with acetone and dried in vacuo (0.09 g, yield 29%). Mp = 456–458 K.

### 1.1. Refinement

Reflections 0 1 1 and 0 1 2 were affected by the beam stop and were omitted from the refinement.The H atoms were positioned geometrically and refined using a riding model, with C—H = 0.95 with *U*_iso_(H) = 1.2 *U*_eq_(C) for H atoms on C(*sp*^2^) and 0.98 Å with *U*_iso_(H) = 1.5 *U*_eq_(C) for H atoms on C(*sp*^3^). The aceto­nitrile exhibits disorder over two different orientations. The occupancies refined to 0.629 (15) and 0.371 (15).

### 2. Results and discussion

Copper(I) complexes have many applications. Many of these complexes have been of increasing inter­est due to the variety of their structures and their similarity to metallo­thio­neins. The role of copper(I) is evident in several biologically important reactions, such as a di­oxy­gen carrier and models for several enzymes (Krupanidhi *et al.*, 2008). On the other hand, these compounds have been reported to be luminescent (Aslanidis *et al.*, 2010; Gallego *et al.*, 2012) and exhibit corrosion inhibiting properties (Tian *et al.*, 2004). Herein, the title complex was prepared by reacting copper (I) iodide and tri­phenyl­phosphane (PPh_3_), followed by the addition of 5-amino-1,3,4-thia­diazole-2-thiol (ATM) in aceto­nitrile solvent. An unexpexted complex [CuI(C_18_H_15_P)_2_(CH_3_CN)] was formed in the absence of ATM in low yield (29%) (Fig.1). The coordination environment around the Cu^I^ ion is a distorted tetra­hedral geometry fromed by two P atoms of two tri­phenyl­phosphine ligands, one N atom of disordered aceto­nitrile ligand and one iodide atom. The occupancies of the disorder sites of the aceto­nitrile ligand refined to 0.629 (15) and 0.371 (15). The Cu1—N1 bond distance of 2.055 (10) Å is slightly longer than that found in for example [Cu(C_15_H_4_BF_18_N_6_)(C_2_H_3_N)], which is 1.888 (3) Å (Balili & Pintauer, 2007). The aceto­nitrile ligand is almost linear with an N—C—C angle of 177.4 (14)° [or 174 (3)° for the minor component of disorder]. The typical value for an aceto­nitrile ligand, as for the [Cu(CH_3_CN)_4_]^+^ cation (Royappa *et al.*, 2013) are angles in the range 178.4 (3)-179 (3)°. In the crystal, the molecules are connected *via* weak C2—H2*A*···I1^i^ inter­actions, forming a one-dimensional chain along the *a*-axis direction (Fig.2).

### Crystal data

**Table:**

[CuI(C_2_H_3_N)(C_18_H_15_P)_2_]	*F*(000) = 1520
*M**_r_* = 756.03	*D*_x_ = 1.443 Mg m^−^^3^
Monoclinic, *P*2_1_/*n*	Mo *K*α radiation, λ = 0.71073 Å
*a* = 9.2547 (3) Å	Cell parameters from 7884 reflections
*b* = 19.3814 (6) Å	θ = 2.4–31.2°
*c* = 19.4249 (6) Å	µ = 1.63 mm^−^^1^
β = 93.043 (1)°	*T* = 100 K
*V* = 3479.31 (19) Å^3^	Plate, colourless
*Z* = 4	0.33 × 0.22 × 0.09 mm

### Data collection

**Table:**

Bruker AXS SMART APEX CCD diffractometer	8394 reflections with *I* > 2σ(*I*)
Radiation source: fine focus sealed tube	*R*_int_ = 0.025
ω and φ scans	θ_max_ = 31.1°, θ_min_ = 2.4°
Absorption correction: multi-scan (*SADABS*; Bruker, 2012)	*h* = −12→13
*T*_min_ = 0.600, *T*_max_ = 0.746	*k* = −27→28
20389 measured reflections	*l* = −26→21
10066 independent reflections	

### Refinement

**Table:**

Refinement on *F*^2^	Primary atom site location: structure-invariant direct methods
Least-squares matrix: full	Secondary atom site location: difference Fourier map
*R*[*F*^2^ > 2σ(*F*^2^)] = 0.036	Hydrogen site location: inferred from neighbouring sites
*wR*(*F*^2^) = 0.086	H-atom parameters constrained
*S* = 1.09	*w* = 1/[σ^2^(*F*_o_^2^) + (0.0383*P*)^2^ + 1.2024*P*] where *P* = (*F*_o_^2^ + 2*F*_c_^2^)/3
10066 reflections	(Δ/σ)_max_ = 0.002
400 parameters	Δρ_max_ = 1.59 e Å^−^^3^
3 restraints	Δρ_min_ = −0.52 e Å^−^^3^

### Special details

**Table:**

Geometry. All e.s.d.'s (except the e.s.d. in the dihedral angle between two l.s. planes) are estimated using the full covariance matrix. The cell e.s.d.'s are taken into account individually in the estimation of e.s.d.'s in distances, angles and torsion angles; correlations between e.s.d.'s in cell parameters are only used when they are defined by crystal symmetry. An approximate (isotropic) treatment of cell e.s.d.'s is used for estimating e.s.d.'s involving l.s. planes.

### Fractional atomic coordinates and isotropic or equivalent isotropic displacement parameters (Å^2^)

**Table:**

	*x*	*y*	*z*	*U*_iso_*/*U*_eq_	Occ. (<1)
I1	0.96840 (2)	0.74638 (2)	0.25147 (2)	0.01967 (5)	
Cu1	1.12630 (3)	0.86273 (2)	0.25454 (2)	0.01449 (6)	
P1	1.08578 (6)	0.91578 (3)	0.35789 (3)	0.01380 (11)	
P2	1.07803 (6)	0.91388 (3)	0.14896 (3)	0.01330 (11)	
N1	1.3267 (11)	0.8180 (8)	0.2489 (11)	0.0200 (17)	0.629 (15)
C1	1.4315 (9)	0.7867 (5)	0.2453 (5)	0.0213 (15)	0.629 (15)
C2	1.5653 (8)	0.7464 (5)	0.2371 (5)	0.051 (2)	0.629 (15)
H2A	1.6412	0.7629	0.2701	0.076*	0.629 (15)
H2B	1.5969	0.7522	0.1901	0.076*	0.629 (15)
H2C	1.5463	0.6975	0.2457	0.076*	0.629 (15)
N1B	1.336 (2)	0.8304 (15)	0.254 (2)	0.0200 (17)	0.371 (15)
C1B	1.4352 (17)	0.8042 (8)	0.2331 (10)	0.0213 (15)	0.371 (15)
C2B	1.5702 (15)	0.7720 (9)	0.2124 (8)	0.051 (2)	0.371 (15)
H2D	1.6506	0.7868	0.2439	0.076*	0.371 (15)
H2E	1.5892	0.7861	0.1653	0.076*	0.371 (15)
H2F	1.5609	0.7217	0.2143	0.076*	0.371 (15)
C11	1.1283 (2)	0.86224 (11)	0.43498 (11)	0.0162 (4)	
C12	1.0491 (3)	0.86457 (12)	0.49505 (12)	0.0221 (5)	
H12	0.9630	0.8912	0.4956	0.027*	
C13	1.0978 (3)	0.82739 (14)	0.55452 (12)	0.0281 (6)	
H13	1.0438	0.8287	0.5948	0.034*	
C14	1.2247 (3)	0.78884 (14)	0.55407 (13)	0.0295 (6)	
H14	1.2581	0.7643	0.5941	0.035*	
C15	1.3029 (3)	0.78641 (14)	0.49446 (13)	0.0270 (5)	
H15	1.3898	0.7603	0.4943	0.032*	
C16	1.2542 (3)	0.82223 (12)	0.43488 (12)	0.0210 (5)	
H16	1.3069	0.8193	0.3943	0.025*	
C21	1.1866 (2)	0.99578 (11)	0.37978 (11)	0.0151 (4)	
C22	1.2632 (2)	1.00580 (12)	0.44391 (11)	0.0174 (4)	
H22	1.2554	0.9729	0.4798	0.021*	
C23	1.3505 (3)	1.06426 (12)	0.45463 (12)	0.0209 (5)	
H23	1.4025	1.0705	0.4977	0.025*	
C24	1.3619 (3)	1.11372 (12)	0.40247 (13)	0.0224 (5)	
H24	1.4226	1.1528	0.4101	0.027*	
C25	1.2838 (3)	1.10554 (12)	0.33921 (13)	0.0236 (5)	
H25	1.2888	1.1397	0.3043	0.028*	
C26	1.1982 (3)	1.04656 (12)	0.32783 (12)	0.0205 (5)	
H26	1.1471	1.0405	0.2845	0.025*	
C31	0.8948 (2)	0.93943 (12)	0.36381 (11)	0.0174 (4)	
C32	0.7922 (3)	0.88569 (13)	0.36523 (12)	0.0219 (5)	
H32	0.8233	0.8391	0.3699	0.026*	
C33	0.6451 (3)	0.90124 (15)	0.35968 (13)	0.0270 (5)	
H33	0.5760	0.8651	0.3614	0.032*	
C34	0.5982 (3)	0.96955 (16)	0.35156 (13)	0.0322 (6)	
H34	0.4977	0.9795	0.3464	0.039*	
C35	0.6980 (3)	1.02267 (16)	0.35101 (15)	0.0347 (6)	
H35	0.6659	1.0691	0.3464	0.042*	
C36	0.8460 (3)	1.00806 (13)	0.35728 (13)	0.0266 (5)	
H36	0.9142	1.0447	0.3571	0.032*	
C41	0.8856 (2)	0.91370 (11)	0.12315 (11)	0.0164 (4)	
C42	0.8312 (2)	0.89980 (12)	0.05587 (12)	0.0198 (5)	
H42	0.8960	0.8906	0.0207	0.024*	
C43	0.6829 (3)	0.89942 (14)	0.04044 (13)	0.0254 (5)	
H43	0.6463	0.8898	−0.0052	0.031*	
C44	0.5882 (3)	0.91312 (18)	0.09190 (14)	0.0394 (8)	
H44	0.4867	0.9129	0.0814	0.047*	
C45	0.6415 (3)	0.9272 (2)	0.15896 (15)	0.0615 (12)	
H45	0.5762	0.9367	0.1939	0.074*	
C46	0.7893 (3)	0.9275 (2)	0.17500 (14)	0.0446 (9)	
H46	0.8252	0.9369	0.2208	0.054*	
C51	1.1667 (2)	0.87560 (11)	0.07426 (11)	0.0145 (4)	
C52	1.2127 (2)	0.91528 (12)	0.01858 (11)	0.0177 (4)	
H52	1.1952	0.9636	0.0176	0.021*	
C53	1.2845 (3)	0.88405 (13)	−0.03579 (12)	0.0218 (5)	
H53	1.3162	0.9114	−0.0727	0.026*	
C54	1.3089 (3)	0.81271 (13)	−0.03523 (13)	0.0256 (5)	
H54	1.3578	0.7915	−0.0715	0.031*	
C55	1.2604 (3)	0.77261 (13)	0.01957 (15)	0.0290 (6)	
H55	1.2746	0.7241	0.0195	0.035*	
C56	1.1915 (3)	0.80371 (12)	0.07425 (12)	0.0205 (5)	
H56	1.1613	0.7763	0.1114	0.025*	
C61	1.1305 (3)	1.00548 (11)	0.14349 (11)	0.0174 (4)	
C62	1.2766 (3)	1.02147 (13)	0.15747 (13)	0.0253 (5)	
H62	1.3441	0.9853	0.1669	0.030*	
C63	1.3247 (3)	1.09004 (14)	0.15778 (13)	0.0320 (6)	
H63	1.4244	1.1000	0.1668	0.038*	
C64	1.2260 (4)	1.14374 (14)	0.14489 (14)	0.0426 (8)	
H64	1.2581	1.1903	0.1456	0.051*	
C65	1.0822 (4)	1.12864 (14)	0.13109 (15)	0.0451 (9)	
H65	1.0150	1.1650	0.1218	0.054*	
C66	1.0337 (3)	1.05963 (13)	0.13057 (13)	0.0295 (6)	
H66	0.9339	1.0500	0.1213	0.035*	

### Atomic displacement parameters (Å^2^)

**Table:**

	*U*^11^	*U*^22^	*U*^33^	*U*^12^	*U*^13^	*U*^23^
I1	0.01685 (8)	0.01524 (7)	0.02709 (9)	0.00172 (5)	0.00276 (6)	−0.00058 (5)
Cu1	0.01291 (12)	0.01785 (13)	0.01256 (13)	0.00217 (10)	−0.00077 (9)	−0.00019 (10)
P1	0.0128 (3)	0.0166 (3)	0.0120 (3)	−0.0004 (2)	0.00034 (19)	−0.0007 (2)
P2	0.0130 (2)	0.0149 (2)	0.0119 (3)	0.0020 (2)	−0.00034 (19)	−0.00071 (19)
N1	0.0145 (15)	0.029 (6)	0.016 (3)	−0.002 (2)	−0.0040 (17)	−0.009 (3)
C1	0.0144 (12)	0.033 (5)	0.016 (4)	0.000 (3)	−0.0013 (17)	−0.004 (3)
C2	0.0310 (19)	0.076 (6)	0.046 (5)	0.024 (3)	0.009 (3)	−0.023 (3)
N1B	0.0145 (15)	0.029 (6)	0.016 (3)	−0.002 (2)	−0.0040 (17)	−0.009 (3)
C1B	0.0144 (12)	0.033 (5)	0.016 (4)	0.000 (3)	−0.0013 (17)	−0.004 (3)
C2B	0.0310 (19)	0.076 (6)	0.046 (5)	0.024 (3)	0.009 (3)	−0.023 (3)
C11	0.0180 (10)	0.0168 (10)	0.0137 (10)	−0.0029 (8)	−0.0018 (8)	−0.0006 (8)
C12	0.0242 (12)	0.0250 (12)	0.0174 (11)	0.0007 (10)	0.0033 (9)	0.0010 (9)
C13	0.0363 (15)	0.0340 (14)	0.0143 (11)	0.0026 (12)	0.0042 (10)	0.0033 (10)
C14	0.0411 (16)	0.0304 (13)	0.0164 (12)	0.0020 (12)	−0.0036 (10)	0.0042 (10)
C15	0.0280 (13)	0.0280 (13)	0.0247 (13)	0.0074 (10)	−0.0022 (10)	0.0004 (10)
C16	0.0225 (12)	0.0225 (11)	0.0179 (11)	0.0017 (9)	0.0017 (9)	−0.0012 (9)
C21	0.0137 (10)	0.0170 (10)	0.0148 (10)	0.0006 (8)	0.0018 (8)	−0.0024 (8)
C22	0.0178 (10)	0.0217 (11)	0.0126 (10)	0.0007 (9)	0.0014 (8)	−0.0013 (8)
C23	0.0222 (12)	0.0239 (11)	0.0163 (11)	−0.0006 (9)	−0.0006 (9)	−0.0072 (9)
C24	0.0240 (12)	0.0189 (11)	0.0246 (12)	−0.0030 (9)	0.0024 (9)	−0.0071 (9)
C25	0.0298 (13)	0.0193 (11)	0.0218 (12)	−0.0028 (10)	0.0022 (10)	0.0018 (9)
C26	0.0233 (12)	0.0220 (11)	0.0158 (11)	−0.0038 (9)	−0.0014 (9)	0.0001 (9)
C31	0.0151 (10)	0.0249 (11)	0.0120 (10)	0.0018 (9)	0.0009 (8)	−0.0032 (8)
C32	0.0181 (11)	0.0299 (12)	0.0181 (11)	−0.0025 (9)	0.0038 (9)	−0.0061 (9)
C33	0.0168 (11)	0.0445 (15)	0.0200 (12)	−0.0054 (11)	0.0045 (9)	−0.0104 (11)
C34	0.0160 (12)	0.0541 (18)	0.0266 (13)	0.0082 (12)	0.0019 (10)	−0.0108 (12)
C35	0.0232 (13)	0.0393 (15)	0.0417 (16)	0.0143 (12)	0.0044 (11)	−0.0047 (13)
C36	0.0206 (12)	0.0276 (13)	0.0317 (14)	0.0030 (10)	0.0031 (10)	−0.0031 (10)
C41	0.0131 (10)	0.0199 (10)	0.0161 (10)	0.0038 (8)	−0.0011 (8)	0.0012 (8)
C42	0.0156 (10)	0.0253 (11)	0.0188 (11)	−0.0002 (9)	0.0031 (8)	−0.0033 (9)
C43	0.0178 (11)	0.0385 (14)	0.0196 (12)	−0.0001 (10)	−0.0034 (9)	0.0001 (10)
C44	0.0123 (11)	0.080 (2)	0.0255 (14)	0.0078 (13)	−0.0026 (10)	0.0041 (14)
C45	0.0212 (14)	0.144 (4)	0.0197 (14)	0.0259 (19)	0.0038 (11)	−0.0005 (19)
C46	0.0199 (13)	0.101 (3)	0.0130 (12)	0.0221 (15)	−0.0012 (10)	−0.0020 (14)
C51	0.0111 (9)	0.0192 (10)	0.0129 (10)	0.0010 (8)	−0.0021 (7)	−0.0027 (8)
C52	0.0170 (11)	0.0203 (11)	0.0156 (10)	0.0037 (8)	−0.0016 (8)	0.0001 (8)
C53	0.0207 (11)	0.0290 (12)	0.0157 (11)	0.0017 (10)	0.0023 (9)	0.0012 (9)
C54	0.0235 (12)	0.0322 (13)	0.0218 (12)	0.0058 (10)	0.0078 (9)	−0.0065 (10)
C55	0.0318 (14)	0.0198 (11)	0.0364 (15)	0.0071 (11)	0.0110 (11)	−0.0055 (11)
C56	0.0218 (11)	0.0185 (11)	0.0219 (12)	0.0014 (9)	0.0067 (9)	−0.0009 (9)
C61	0.0259 (12)	0.0163 (10)	0.0101 (10)	−0.0019 (9)	0.0007 (8)	−0.0019 (8)
C62	0.0296 (13)	0.0216 (12)	0.0255 (13)	−0.0034 (10)	0.0068 (10)	−0.0059 (10)
C63	0.0448 (17)	0.0303 (14)	0.0213 (13)	−0.0183 (12)	0.0069 (11)	−0.0067 (10)
C64	0.081 (2)	0.0220 (13)	0.0235 (14)	−0.0137 (14)	−0.0138 (14)	0.0016 (11)
C65	0.076 (2)	0.0176 (12)	0.0382 (17)	0.0061 (14)	−0.0297 (16)	0.0008 (11)
C66	0.0391 (15)	0.0213 (12)	0.0261 (13)	0.0076 (11)	−0.0159 (11)	−0.0030 (10)

### Geometric parameters (Å, º)

**Table:**

I1—Cu1	2.6861 (3)	C31—C32	1.410 (3)
Cu1—N1B	2.037 (18)	C32—C33	1.393 (3)
Cu1—N1	2.055 (10)	C32—H32	0.9500
Cu1—P2	2.3003 (6)	C33—C34	1.400 (4)
Cu1—P1	2.3038 (6)	C33—H33	0.9500
P1—C31	1.836 (2)	C34—C35	1.384 (4)
P1—C11	1.847 (2)	C34—H34	0.9500
P1—C21	1.847 (2)	C35—C36	1.397 (3)
P2—C41	1.824 (2)	C35—H35	0.9500
P2—C61	1.845 (2)	C36—H36	0.9500
P2—C51	1.859 (2)	C41—C42	1.402 (3)
N1—C1	1.149 (9)	C41—C46	1.406 (3)
C1—C2	1.479 (7)	C42—C43	1.389 (3)
C2—H2A	0.9800	C42—H42	0.9500
C2—H2B	0.9800	C43—C44	1.390 (4)
C2—H2C	0.9800	C43—H43	0.9500
N1B—C1B	1.148 (13)	C44—C45	1.395 (4)
C1B—C2B	1.471 (12)	C44—H44	0.9500
C2B—H2D	0.9800	C45—C46	1.386 (4)
C2B—H2E	0.9800	C45—H45	0.9500
C2B—H2F	0.9800	C46—H46	0.9500
C11—C16	1.400 (3)	C51—C52	1.411 (3)
C11—C12	1.411 (3)	C51—C56	1.412 (3)
C12—C13	1.414 (3)	C52—C53	1.413 (3)
C12—H12	0.9500	C52—H52	0.9500
C13—C14	1.393 (4)	C53—C54	1.401 (3)
C13—H13	0.9500	C53—H53	0.9500
C14—C15	1.399 (4)	C54—C55	1.410 (4)
C14—H14	0.9500	C54—H54	0.9500
C15—C16	1.403 (3)	C55—C56	1.404 (3)
C15—H15	0.9500	C55—H55	0.9500
C16—H16	0.9500	C56—H56	0.9500
C21—C22	1.414 (3)	C61—C66	1.394 (3)
C21—C26	1.418 (3)	C61—C62	1.399 (3)
C22—C23	1.401 (3)	C62—C63	1.401 (3)
C22—H22	0.9500	C62—H62	0.9500
C23—C24	1.403 (3)	C63—C64	1.398 (4)
C23—H23	0.9500	C63—H63	0.9500
C24—C25	1.401 (3)	C64—C65	1.375 (5)
C24—H24	0.9500	C64—H64	0.9500
C25—C26	1.402 (3)	C65—C66	1.411 (4)
C25—H25	0.9500	C65—H65	0.9500
C26—H26	0.9500	C66—H66	0.9500
C31—C36	1.408 (3)		
			
N1B—Cu1—P2	105.7 (12)	C36—C31—C32	119.1 (2)
N1—Cu1—P2	105.5 (7)	C36—C31—P1	122.36 (18)
N1B—Cu1—P1	109.7 (9)	C32—C31—P1	117.91 (17)
N1—Cu1—P1	115.1 (5)	C33—C32—C31	119.6 (2)
P2—Cu1—P1	123.48 (2)	C33—C32—H32	120.2
N1B—Cu1—I1	105.0 (8)	C31—C32—H32	120.2
N1—Cu1—I1	97.8 (4)	C32—C33—C34	120.6 (2)
P2—Cu1—I1	105.208 (17)	C32—C33—H33	119.7
P1—Cu1—I1	106.291 (17)	C34—C33—H33	119.7
C31—P1—C11	104.69 (10)	C35—C34—C33	120.1 (2)
C31—P1—C21	104.61 (10)	C35—C34—H34	120.0
C11—P1—C21	101.64 (10)	C33—C34—H34	120.0
C31—P1—Cu1	111.59 (7)	C34—C35—C36	120.0 (3)
C11—P1—Cu1	114.73 (7)	C34—C35—H35	120.0
C21—P1—Cu1	118.14 (7)	C36—C35—H35	120.0
C41—P2—C61	104.01 (10)	C35—C36—C31	120.5 (2)
C41—P2—C51	104.46 (10)	C35—C36—H36	119.8
C61—P2—C51	102.15 (10)	C31—C36—H36	119.8
C41—P2—Cu1	112.63 (7)	C42—C41—C46	119.6 (2)
C61—P2—Cu1	115.26 (7)	C42—C41—P2	123.63 (17)
C51—P2—Cu1	116.81 (7)	C46—C41—P2	116.72 (17)
C1—N1—Cu1	173.0 (12)	C43—C42—C41	120.3 (2)
N1—C1—C2	177.4 (14)	C43—C42—H42	119.9
C1—C2—H2A	109.5	C41—C42—H42	119.9
C1—C2—H2B	109.5	C42—C43—C44	119.8 (2)
H2A—C2—H2B	109.5	C42—C43—H43	120.1
C1—C2—H2C	109.5	C44—C43—H43	120.1
H2A—C2—H2C	109.5	C43—C44—C45	120.2 (2)
H2B—C2—H2C	109.5	C43—C44—H44	119.9
C1B—N1B—Cu1	157 (3)	C45—C44—H44	119.9
N1B—C1B—C2B	174 (3)	C46—C45—C44	120.4 (3)
C1B—C2B—H2D	109.5	C46—C45—H45	119.8
C1B—C2B—H2E	109.5	C44—C45—H45	119.8
H2D—C2B—H2E	109.5	C45—C46—C41	119.6 (2)
C1B—C2B—H2F	109.5	C45—C46—H46	120.2
H2D—C2B—H2F	109.5	C41—C46—H46	120.2
H2E—C2B—H2F	109.5	C52—C51—C56	118.8 (2)
C16—C11—C12	119.2 (2)	C52—C51—P2	122.99 (16)
C16—C11—P1	116.83 (17)	C56—C51—P2	118.18 (17)
C12—C11—P1	123.72 (17)	C51—C52—C53	120.8 (2)
C11—C12—C13	120.2 (2)	C51—C52—H52	119.6
C11—C12—H12	119.9	C53—C52—H52	119.6
C13—C12—H12	119.9	C54—C53—C52	119.9 (2)
C14—C13—C12	120.0 (2)	C54—C53—H53	120.0
C14—C13—H13	120.0	C52—C53—H53	120.0
C12—C13—H13	120.0	C53—C54—C55	119.5 (2)
C13—C14—C15	119.8 (2)	C53—C54—H54	120.3
C13—C14—H14	120.1	C55—C54—H54	120.3
C15—C14—H14	120.1	C56—C55—C54	120.7 (2)
C14—C15—C16	120.6 (2)	C56—C55—H55	119.7
C14—C15—H15	119.7	C54—C55—H55	119.7
C16—C15—H15	119.7	C55—C56—C51	120.3 (2)
C11—C16—C15	120.3 (2)	C55—C56—H56	119.9
C11—C16—H16	119.9	C51—C56—H56	119.9
C15—C16—H16	119.9	C66—C61—C62	118.3 (2)
C22—C21—C26	118.6 (2)	C66—C61—P2	124.48 (19)
C22—C21—P1	122.79 (17)	C62—C61—P2	117.15 (18)
C26—C21—P1	118.38 (16)	C61—C62—C63	120.9 (3)
C23—C22—C21	120.0 (2)	C61—C62—H62	119.5
C23—C22—H22	120.0	C63—C62—H62	119.5
C21—C22—H22	120.0	C64—C63—C62	120.1 (3)
C22—C23—C24	120.7 (2)	C64—C63—H63	120.0
C22—C23—H23	119.7	C62—C63—H63	120.0
C24—C23—H23	119.7	C65—C64—C63	119.5 (3)
C25—C24—C23	120.1 (2)	C65—C64—H64	120.3
C25—C24—H24	120.0	C63—C64—H64	120.3
C23—C24—H24	120.0	C64—C65—C66	120.5 (3)
C26—C25—C24	119.4 (2)	C64—C65—H65	119.7
C26—C25—H25	120.3	C66—C65—H65	119.7
C24—C25—H25	120.3	C61—C66—C65	120.7 (3)
C25—C26—C21	121.2 (2)	C61—C66—H66	119.6
C25—C26—H26	119.4	C65—C66—H66	119.6
C21—C26—H26	119.4		
			
C31—P1—C11—C16	163.69 (17)	C61—P2—C41—C42	95.7 (2)
C21—P1—C11—C16	−87.64 (18)	C51—P2—C41—C42	−11.1 (2)
Cu1—P1—C11—C16	41.04 (19)	Cu1—P2—C41—C42	−138.85 (18)
C31—P1—C11—C12	−22.3 (2)	C61—P2—C41—C46	−85.3 (2)
C21—P1—C11—C12	86.4 (2)	C51—P2—C41—C46	168.0 (2)
Cu1—P1—C11—C12	−144.94 (17)	Cu1—P2—C41—C46	40.2 (2)
C16—C11—C12—C13	0.6 (3)	C46—C41—C42—C43	−0.2 (4)
P1—C11—C12—C13	−173.25 (19)	P2—C41—C42—C43	178.85 (19)
C11—C12—C13—C14	0.6 (4)	C41—C42—C43—C44	0.2 (4)
C12—C13—C14—C15	−0.7 (4)	C42—C43—C44—C45	0.0 (5)
C13—C14—C15—C16	−0.3 (4)	C43—C44—C45—C46	−0.2 (6)
C12—C11—C16—C15	−1.7 (3)	C44—C45—C46—C41	0.3 (6)
P1—C11—C16—C15	172.63 (19)	C42—C41—C46—C45	−0.1 (5)
C14—C15—C16—C11	1.5 (4)	P2—C41—C46—C45	−179.2 (3)
C31—P1—C21—C22	105.21 (19)	C41—P2—C51—C52	88.90 (19)
C11—P1—C21—C22	−3.5 (2)	C61—P2—C51—C52	−19.2 (2)
Cu1—P1—C21—C22	−130.00 (17)	Cu1—P2—C51—C52	−145.96 (15)
C31—P1—C21—C26	−80.63 (19)	C41—P2—C51—C56	−92.43 (18)
C11—P1—C21—C26	170.64 (18)	C61—P2—C51—C56	159.43 (17)
Cu1—P1—C21—C26	44.2 (2)	Cu1—P2—C51—C56	32.71 (19)
C26—C21—C22—C23	−1.2 (3)	C56—C51—C52—C53	−1.0 (3)
P1—C21—C22—C23	172.94 (17)	P2—C51—C52—C53	177.62 (17)
C21—C22—C23—C24	0.7 (3)	C51—C52—C53—C54	0.9 (3)
C22—C23—C24—C25	1.0 (4)	C52—C53—C54—C55	0.4 (4)
C23—C24—C25—C26	−2.0 (4)	C53—C54—C55—C56	−1.6 (4)
C24—C25—C26—C21	1.5 (4)	C54—C55—C56—C51	1.5 (4)
C22—C21—C26—C25	0.1 (3)	C52—C51—C56—C55	−0.1 (3)
P1—C21—C26—C25	−174.27 (19)	P2—C51—C56—C55	−178.85 (19)
C11—P1—C31—C36	129.7 (2)	C41—P2—C61—C66	7.0 (2)
C21—P1—C31—C36	23.2 (2)	C51—P2—C61—C66	115.5 (2)
Cu1—P1—C31—C36	−105.69 (19)	Cu1—P2—C61—C66	−116.78 (19)
C11—P1—C31—C32	−59.41 (19)	C41—P2—C61—C62	−177.03 (18)
C21—P1—C31—C32	−165.90 (17)	C51—P2—C61—C62	−68.54 (19)
Cu1—P1—C31—C32	65.25 (18)	Cu1—P2—C61—C62	59.18 (19)
C36—C31—C32—C33	0.5 (3)	C66—C61—C62—C63	−0.7 (4)
P1—C31—C32—C33	−170.79 (18)	P2—C61—C62—C63	−176.87 (19)
C31—C32—C33—C34	1.1 (4)	C61—C62—C63—C64	0.8 (4)
C32—C33—C34—C35	−1.9 (4)	C62—C63—C64—C65	−0.8 (4)
C33—C34—C35—C36	1.2 (4)	C63—C64—C65—C66	0.6 (5)
C34—C35—C36—C31	0.4 (4)	C62—C61—C66—C65	0.5 (4)
C32—C31—C36—C35	−1.2 (4)	P2—C61—C66—C65	176.4 (2)
P1—C31—C36—C35	169.7 (2)	C64—C65—C66—C61	−0.5 (5)

### Hydrogen-bond geometry (Å, º)

**Table:**

*D*—H···*A*	*D*—H	H···*A*	*D*···*A*	*D*—H···*A*
C2—H2*A*···I1^i^	0.98	3.09	3.727 (8)	124

Symmetry code: (i) *x*+1, *y*, *z*.
